# A systematic review reporting quality of radiomics research in neuro-oncology: toward clinical utility and quality improvement using high-dimensional imaging features

**DOI:** 10.1186/s12885-019-6504-5

**Published:** 2020-01-10

**Authors:** Ji Eun Park, Ho Sung Kim, Donghyun Kim, Seo Young Park, Jung Youn Kim, Se Jin Cho, Jeong Hoon Kim

**Affiliations:** 1 Department of Radiology and Research Institute of Radiology, University of Ulsan College of Medicine, Asan Medical Center, 43 Olympic-ro 88, Songpa-Gu, Seoul, 05505 South Korea; 2 Department of Radiology, Inje University Busan Paik Hospital, Busan, South Korea; 3 Department of Clinical Epidemiology and Biostatistics, University of Ulsan College of Medicine, Asan Medical Center, Seoul, South Korea; 4 Department of Radiology, Kangbuk Samsung Medical Center, Seoul, South Korea; 5 Department of Neurosurgery, University of Ulsan College of Medicine, Asan Medical Center, Seoul, South Korea

**Keywords:** Radiomics, Neuro-oncology, Quality, Score

## Abstract

**Background:**

To evaluate radiomics analysis in neuro-oncologic studies according to a radiomics quality score (RQS) system to find room for improvement in clinical use.

**Methods:**

Pubmed and Embase were searched up the terms radiomics or radiogenomics and gliomas or glioblastomas until February 2019. From 189 articles, 51 original research articles reporting the diagnostic, prognostic, or predictive utility were selected. The quality of the methodology was evaluated according to the RQS. The adherence rates for the six key domains were evaluated: image protocol and reproducibility, feature reduction and validation, biologic/clinical utility, performance index, a high level of evidence, and open science. Subgroup analyses for journal type (imaging vs. clinical) and biomarker (diagnostic vs. prognostic/predictive) were performed.

**Results:**

The median RQS was 11 out of 36 and adherence rate was 37.1%. Only 29.4% performed external validation. The adherence rate was high for reporting imaging protocol (100%), feature reduction (94.1%), and discrimination statistics (96.1%), but low for conducting test-retest analysis (2%), prospective study (3.9%), demonstrating potential clinical utility (2%), and open science (5.9%). None of the studies conducted a phantom study or cost-effectiveness analysis. Prognostic/predictive studies received higher score than diagnostic studies in comparison to gold standard (*P* < .001), use of calibration (*P* = .02), and cut-off analysis (*P* = .001).

**Conclusions:**

The quality of reporting of radiomics studies in neuro-oncology is currently insufficient. Validation is necessary using external dataset, and improvements need to be made to feature reproducibility, demonstrating clinical utility, pursuits of a higher level of evidence, and open science.

## Background

Radiomics is a powerful tool for developing and testing medical hypotheses, involving the use of high-dimensional quantitative imaging features for predictive purposes. The extraction of high-throughput quantitative features and the use of sophisticated bioinformatics tools enables the development of models with potential diagnostic, prognostic, or predictive utility in cancer studies [1–3]. In the field of neuro-oncology, a large number of radiomics studies have demonstrated their diagnostic, prognostic, and predictive use in differential diagnosis [4, 5], molecular classification [6–8], survival analysis [9, 10], and treatment response to antiangiogenic treatment [11].

Although radiomics research shows great potential, its current use is rather confined to the academic literature, without real-world clinical applications. This is in part due to a lack of efficient and effective strategies for biomarker translation [12], which hampers the effective development of radiomics as an imaging biomarker to cross the ‘translational gap’ for use in guiding clinical decisions [13, 14]. A standardized evaluation of the performance, reproducibility, and/or clinical utility of radiomics biomarkers is needed; with regard to the great need for qualified reporting, a system of metrics to determine the validity and completeness of radiomics studies was developed by Lambin et al. [2] in the form of the radiomics quality score (RQS). The RQS is comprised of 16 components, chosen to emulate the Transparent Reporting of a multivariable prediction model for Individual Prognosis OR Diagnosis (TRIPOD) initiative [15]. These are applied to a radiomics-specific design that considers high-dimensional data and modeling, and emphasizes clinical adoption of modeling research as in the TRIPOD guidelines.

A subsequent RQS study from the developer [3] reported an average score less than 50% over 41 radiomics studies using various modalities and conditions, including ultrasound, computed tomography, positron emission tomography, and magnetic resonance imaging (MRI). However, the results do not represent the field of neuro-oncology as both disease and imaging modality varies. Most of neuro-oncologic imaging studies are based on MRI, which is particularly challenging in generalizability and robustness in radiomics analysis as it has non-standardized pixel-values and large variations in signal intensities. To our knowledge, the quality of the science and reporting in radiomics research studies in the neuro-oncologic imaging is largely unknown.

In this study, we evaluated the radiomics analysis conducted in previous publications, summarizing six domains from the RQS: image protocol and feature reproducibility, feature reduction and validation, biologic/clinical validation and utility, performance index, high level of evidence, and open science. Our intention was to promote the quality of radiomics research studies as diagnostic, prognostic, and/or predictive biomarkers, to allow radiomics to become a viable tool for medical decision-making by facilitating the combined analysis of clinical data and high-throughput imaging features. The purpose of our study was to evaluate the quality of reporting radiomics-based analysis in neuro-oncologic studies using RQS.

## Materials and methods

### Article search strategy and study selection

**Fig. 1:**
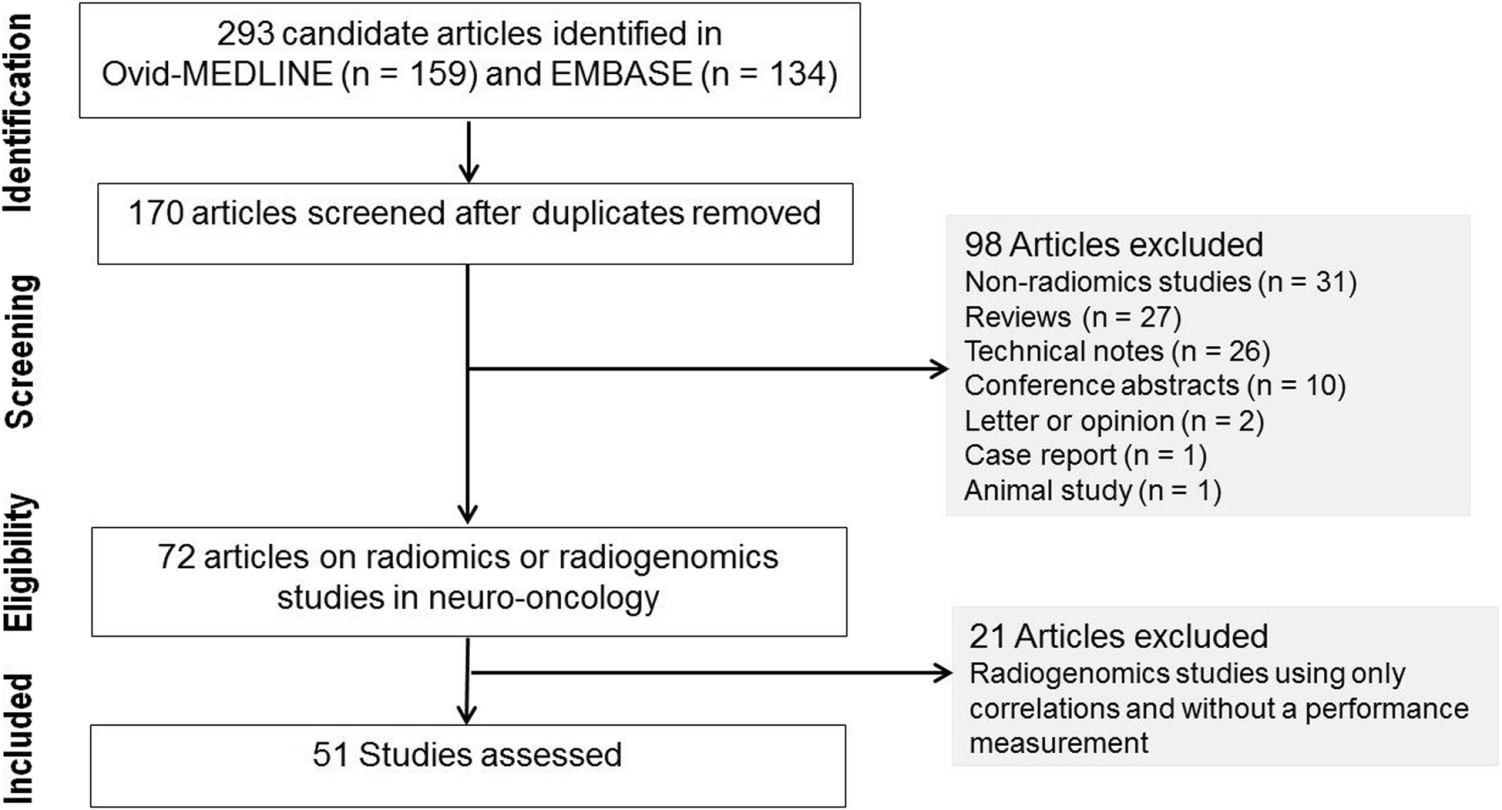
Flow diagram of the study selection process

To identify all potentially relevant original research papers published in the neuro-oncology field, database search was conducted in the MEDLINE (National Center for Biotechnology Information, NCBI) and EMBASE databases up from any time until February 28, 2019. The search terms used to find radiomics studies were “glioma” OR “glioblastoma” AND “radiomic” OR “radiogenomic”. The search identified 293 candidate articles. Retrieved articles were screened for eligibility. After removal of 122 duplicates, screening of the abstracts of the remaining 171 articles was performed. Abstract review further excludes 98 articles for the following reasons: 31 non-radiomics studies, 27 reviews, 26 technical notes, 10 conference abstracts, 2 letter or opinion, 1 case report, and 1 animal study. Full-text reviews of the 73 potentially eligible articles were performed by two experienced reviewer (H.S.K., with 20 years of experience in neuro-oncologic imaging and J.H.K., with 25 years of experience in neurosurgery) selected articles in consensus that tested performance in respect to a diagnostic, prognostic, or predictive biomarker study aspect. Radiogenomics studies searching only for correlations and not containing a performance measurement for a diagnostic, prognostic, or predictive biomarker, were excluded. This process further removed 21 articles as they did not comprise performance tests for diagnostic, prognostic, or predictive utility. Finally, 52 articles were included in the main analysis (Fig. 1).

### Data extraction and analysis

Three reviewers (J.E.P., D.K., who had 5 and 2 years of experience in neuro-oncologic imaging and H.S.K.) evaluated the eligible radiomics performance studies. Before performing their analysis, a seminar was convened in which the reviewers participated, to review and discuss the items listed in the RQS and to ensure they all had a clear knowledge of RQS.

The detailed RQS score with 16 components is defined elsewhere [2] (Additional file 1: Table S1). The reviewers extracted the data using a predetermined RQS evaluation according to six domains. Domain 1 covered protocol quality and reproducibility in image and segmentation: well-documented image protocols (1 point) and/or usage of public image protocols (1 point), multiple segmentations (1 point), phantom study (1 point), and test-retest analysis with imaging at multiple time points (1 point). Domain 2 covered the reporting of feature reduction and validation: feature reduction or adjustment for multiple testing (3 or − 3 points) and validation (− 5 to 5 points). Domain 3 covered the reporting of biological/clinical validation and utility: multivariate analysis with non-radiomics features (1 point), biologic correlates (1 point), comparison to the gold standard (2 points), and potential clinical utility (2 points). Domain 4 covered reporting of the performance index: reporting of discrimination statistics (1 point) with resampling (1 point), calibration statistics (1 point) with resampling (1 point), and application of cut-off analyses (1 point). Domain 5 covered demonstration of a higher level of evidence: by conducting a prospective study (7 points) or cost-effectiveness analysis (2 points). The final domain (domain 6) covered open science, with open availability of source code and data (4 points).

Each article was evaluated by two of the three independent reviewers. Disagreements between any two reviewers were discussed at a research meeting attended by all three reviewers and an additional statistical reviewer. The following topics were, subject to some initial disagreements and were discussed until a consensus was reached.

1) Multiple segmentation (domain 1): when there were two or more readers, the article earned an additional point as segmentation variability was considered. Automatic segmentation using a convolutional neural network or other automatic software earned a point as the method pursued better segmentation reproducibility.

2) Validation (domain 2): a definition of missing validation (− 5 points) was applied when the article performed cross-validation or nested cross-validation using only the training data, as the validation needs to be performed without retraining and without adaptation of the previous cut-off value from the training data.

3) Multivariate analysis with non-radiomics features (domain 3): When the main study endpoint was survival and the selected radiomics features were further correlated with non-radiomic features, i.e. MGMT (O-6-methylguanine-DNA methyltransferase) promoter methylation status, the article earned an additional point. However, if the main outcome was prediction of IDH (isodehydroxygenase) mutation and the radiomics features were selected for IDH mutation only, the article would not gain any additional score, as this would not provide a more holistic radiomics model.

4) Comparison with the gold standard (domain 3): As there is no TNM staging in neuro-oncology, the well-known parameters of age, Karnofsky performance score, extent of resection [16], IDH, or MGMT status were considered as gold standards for survival analysis.

5) Potential clinical utility (domain 3): According to the consensus statement of the FDA-NIH Biomarker Working Group [13], ‘clinical utility’ is thought to be achieved when a biomarker leads to net improvement of health outcomes or provides information useful for prevention, diagnosis, treatment, and management of a disease [13, 17, 18]. For example, a study earned additional points if decision curve analysis was performed and demonstrated net improvement. Discussion of the potential utility of radiomics without proper analysis did not earn additional points.

The multicentricity of data source was further investigated by one reviewer (J.E.P.). The study was evaluated whether the model validation was conducted using external dataset. The multicentricity was evaluated whether the data source was from single center, multi-center, and public data, when constructing the training set. In addition, whether data acquisition was on 1.5 Tesla or 3.0 Tesla magnet was evaluated.

### Statistical analysis

The total RQS score was calculated for each article and for each component. For all included articles, the total RQS score was calculated (score range, − 8–36) and expressed as median and interquartile range.

For the six domains in the RQS (protocol quality and segmentation, feature selection and validation, biologic/clinical validation and utility, model performance index, high level of evidence, and open science and data), basic adherence was assigned when a score of at least 1 point was obtained without minus points. The basic adherence rate was then calculated in a descriptive manner using proportions, and the proportion (%) of articles that fulfilled each reporting domain was determined.

A graphical display for the proportion of studies with a basic adherence rate was adopted from the suggested graphical display for Quality Assessment of Diagnostic Accuracy Studies–2 results [19].

Subgroup analyses were performed to determine whether the reporting quality differed according to intended use (diagnostic or prognostic) and published journal (imaging journal or clinical journal). Before subgroup analysis, the RQS was plotted for each journal to observe whether there was a systematic difference between each journals (Additional file 2: Figure S2), and with no systematic difference being observed between journals, this effect was not considered. The nonparametric Mann-Whitney U test was used to compare the RQS score in each group. All statistical analyses were performed using SPSS (SPSS version 22; SPSS, Chicago, IL) and *R* (R version 3.3.3; R Foundation for Statistical Computing, Vienna, Austria), and a *P* value < .05 was considered statistically significant.

## Results

### Characteristics of Radiomics studies in Neuro-oncology

Fifty-one articles [4–11, 20–61] were finally analyzed (Additional file 3: Table S2). The characteristics of the articles are summarized in Table 1. Most studies included gliomas (90.1%), with either glioblastomas only (47.1%) or lower grade gliomas only (LGG; 29.4%). Other non-glial tumors were studied in 9.8%. The study purposes included molecular or genomic classification (49.0%), survival prediction (25.5%), differential diagnosis of gliomas from non-glial tumors (11.8%), histopathologic grading (9.8%), and assessment of response to treatment (5.9%). One study predicted the occurrence of epilepsy in LGG patients using radiomics analysis [38]. Radiomics analysis was most frequently studied as a diagnostic biomarker (70.6%), followed by use as a prognostic biomarker (25.5%), and as a predictive (7.8%) biomarker. Analysis of the validation methods revealed that external validation was missing in 36 out of 51 studies (70.6%). Among 51 studies, 66.7% ([34/51]) of studies were from single center, 29.4% ([15/51]) studies were from public data (TCIA), and 3.9% ([2/51]) of studies were from multicenter data source. In regard of magnetic strength, 58.8% ([30/51]) of studies utilized 3 Tesla magnet, while 41.2% ([21/51]) of studies utilized both 1.5 Tesla and 3.0 Tesla magnet (also in the Additional file 3: Table S2).

**Table 1:** Characteristics of the 51 neuro-oncologic radiomics studies with study design of diagnostic, prognostic, or predictive biomarker

Article characteristics	Number of articles*
Patient number	153 (standard deviation 82.2; range 32–439)
Journal type	
Clinical journal	20 (39.2)
Imaging journal	31 (60.8)
Study inclusion	
Gliomas	46 (90.1)
*Glioblastoma only*	24 (47.1)
*Lower grade gliomas only*	15 (29.4)
*Gliomas (WHO grade I to IV)*	7 (13.7)
Other tumors	5 (9.8)
Study intent	
Differential diagnosis	6 (11.8)
Histopathological grade	5 (9.8)
Molecular/genomic classification	25† (49.0)
Survival	13† + (25.5)
Response to treatment	3† + (5.9)
Others	1 (2)
Biomarker	
Diagnostic	36† (70.6)
Prognostic	13† + (25.5)
Predictive	4+ (7.8)
External validation	
Yes	15 (29.4)
No	36 (70.6)

*numbers in parentheses are percentages

†Two studies overlap in both molecular classification and survival (diagnostic and prognostic marker)

+ one study overlaps in both prognostic and predictive biomarker

### Basic adherence rate of the reporting quality according to the six key domains

Table 2 summarizes the basic adherence rate of the radiomics studies according to the six key domains. In domain 1, all studies reported well-documented image acquisition protocols or use of publicly available image databases. Multiple segmentations by two readers or automatic segmentation were performed in 14 of the 51 studies (27.4%). Notably, only one study [10] conducted imaging at multiple time points and tested feature robustness. After test-retest analysis, 37.0% of the radiomic features (386 out of the 1043 extracted features in the study) were stable and reproducible [10] over three different sessions on the same machine.

**Table 2:** Basic adherence rate according to the six key domains

	Basic adherence rate
Total 16 items	37.1%
Domain 1: Protocol quality and stability in image and segmentation	32.3%
Protocol quality	51 (100%)
Test-retest	1 (2%)
Phantom study	0 (0%)
Multiple segmentation	14 (27.4%)
Domain 2: Feature selection and validation	81.4%
Feature reduction or adjustment of multiple testing	48 (94.1%)
Validation	35 (68.6%)
Domain 3: Biologic/clinical validation and utility	39.2%
Multivariate analysis with non-radiomics features	32 (62.7%)
Biologic correlates	28 (74.5%)
Comparison to ‘gold standard’	19 (37.2%)
Potential clinical utility	1 (2%)
Domain 4: Model performance index	45.1%
Discrimination statistics	49 (96.1%)
Calibration statistics	7 (13.7%)
Cut-off analysis	13 (25.5%)
Domain 5: High level of evidence	2%
Prospective study	2 (3.9%)
Cost-effective analysis	0 (0%)
Domain 6: Open science and data	3 (5.9%)

In domain 2, most studies adopted appropriate feature reduction or adjustment for multiple testing (48 out of 51, 94.1%). The studies used either false discovery rate with univariate logistic regression or two-sample t-tests (for binary outcomes), and a variety of statistical and machine learning methods such as Least Absolute Shrinkage Selector Operator (LASSO), random forest, recursive feature elimination, and support vector machines. Many of studies performed validation using datasets from the same or a different institute (35 out of 51, 68.6%). Six studies earned the full 5 points for validation [21, 24, 31, 36, 54, 55], using data from three datasets from distinct institutes or public dataset.

In domain 3, many of studies performed multivariate analysis of the radiomics features with non-radiomic features (62.7%), and most of the studies found biological correlates (74.5%) to provide a more holistic model and imply biological relevance. Less than half of the studies (37.2%) compared results with an existing gold standard. By contrast, in terms of clinical utility, only one study [50] analyzed a net improvement in health outcomes using decision curve analysis or other statistical tools.

In domain 4, all studies used discriminative statistics, but two studies [40, 41] provided hazard ratios and *P* values from a log-rank test for survival analysis instead of the C-index.

**Fig. 2:**
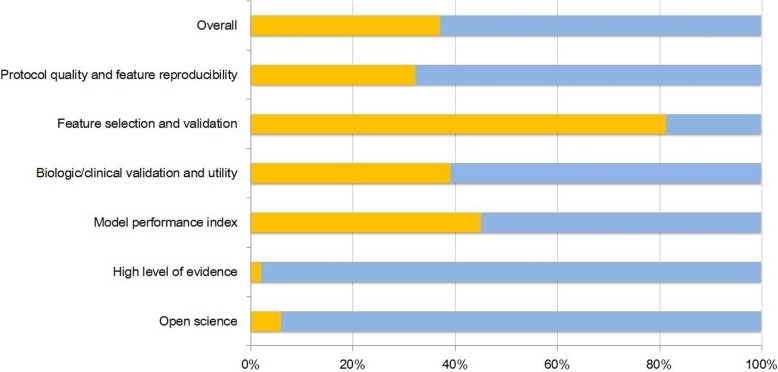
Basic adherence rate of the radiomics quality scores (RQS) of the 51 studies according to the six key domains

Surprisingly, studies were deficient in demonstrating a high level of evidence such as a prospective design or cost-effectiveness analysis. One two studies partly performed validation using a prospective registry [24, 29], but the study per se was conducted in a retrospective manner. For domain 6, only three studies [4, 20, 47] made their code and/or data publicly available. The adherence rate according to the six key domains is shown in Fig. 2.

### Assessment of the Radiomics quality score

The median RQS score of the 51 studies was 11.0, (interquartile range [IQR], 3–12.75), which was 30.5% of the ideal score of 36 (Table 3). The lowest score was − 6 and the highest score was 16 (44.4% of the ideal quality score). Compared with the ideal score, the RQS of the selected studies was lowest in the high level of evidence domain and open science and data domain (0%), followed by biological/clinical validation, and feature reproducibility in image and segmentation.

**Table 3:** Radiomics quality score and subgroup analysis according to journal type and biomarker study design

Radiomics quality score	Median score (Interquartile range)	Clinical (*n* = 20)	Imaging (*n* = 31)	*P*	Diagnostic (*n* = 34)	Prognostic /Predictive† (*n* = 17)	*P*
Total 36 points	11.00 (3–12.5)	11.5 (3–13)	10 (3.5–11.5)	.57	10 (3–11)	12 (6–14)	.07
Domain 1: Protocol quality and stability in image and segmentation (0 to 5 points)	1 (1–2)	1.5 (1–2)	1 (1–2)		1 (1–2)	1 (1–2)	
Protocol quality (2)	1 (1–1.5)	1 (1–1.25)	1 (1–1)	.95	1 (1–1)	1 (1–1.75)	.662
Test-retest (1)	0 (0–0)	0 (0–0)	0 (0–0)	.228	0 (0–0)	0 (0–0)	.169
Phantom study (1)	0 (0–0)	0 (0–0)	0 (0–0)	NA	0 (0–0)	0 (0–0)	NA
Multiple segmentation (1)	0 (0–1)	0 (0–0.25)	0 (0–1)	.76	0 (0–1)	0 (0–1)	.277
Domain 2: Feature selection and validation (−8 to 8 points)	5 (−2–6)	5 (−2–6)	5 (−2–5)		5 (− 2–6)	5 (− 2–6)	
Feature reduction or adjustment of multiple testing (−3 or 3)	3 (3–3)	3 (3–3)	3 (3–3)	.849	3 (3–3)	3 (3–3)	1
Validation (−5, 2, 3, 4, or 5)	2 (− 5–3)	2 (− 5–3)	2 (− 5–2)	.975	2 (−5–3)	2 (− 5–3)	.833
Domain 3: Biologic/clinical validation and utility (0 to 6 points)	2 (1–3)	2 (1–3)	2 (1–2)		2 (1–2)	3 (2–4)	
Non-radiomics features (1)	1 (0–1)	1 (0–1)	1 (0–1)	.799	1 (0–1)	1 (0–1)	.159
Biologic correlates (1)	1 (0–1)	1 (0–1)	1 (0–1)	1	1 (1–1)	1 (0–1)	**.001**
Comparison to ‘gold standard’ (2)	0 (0–2)	0 (0–2)	0 (0–2)	.97	0 (0–0)	2 (2–2)	**<.001**
Potential clinical utility (2)	0 (0–0)	0 (0–0)	0 (0–0)	.44	0 (0–0)	0 (0–0)	.50
Domain 4: Model performance index (0 to 5 points)	2 (1–2)	2 (1–3)	2 (1–3)		2 (1–2)	2 (1–3)	
Discrimination statistics (2)	2 (1–2)	1.5 (1–2)	2 (1–2)	.75	2 (1–2)	2 (1–2)	.99
Calibration statistics (2)	0 (0–0)	0 (0–0)	0 (0–0)	.84	0 (0–0)	0 (0–1)	.**02**
Cut-off analysis (1)	0 (0–0.5)	0 (0–0)	0 (0–1)	.48	0 (0–0)	1 (0–1)	.**001**
Domain 5: High level of evidence (0 to 8 points)	0 (0–0)	0 (0–0)	0 (0–0)		0 (0–0)	0 (0–0)	
Prospective study (7)	0 (0–0)	0 (0–0)	0 (0–0)	.08	0 (0–0)	0 (0–0)	.634
Cost-effective analysis (1)	0 (0–0)	0 (0–0)	0 (0–0)	NA	0 (0–0)	0 (0–0)	NA
Domain 6: Open science and data (0 to 4 points)	0 (0–0)	0 (0–0)	0 (0–0)	**.03**	0 (0–0)	0 (0–0)	1

† Two studies overlap in both diagnostic and prognostic marker were classified as prognostic use

Both feature reduction and validation were missing from the study [51] with the lowest score. Meanwhile, studies with the highest scores [10, 24, 29, 31, 36, 55] earned additional points by using publicly available images from the TCIA (The Cancer Imaging Archive) [24, 31, 36, 55], registry or trial data [24, 29], multiple segmentation [31], test-retest analysis [10], and calculation of calibration index and bootstrapping [10], with all studies fulfilling requirements for image protocol quality, feature reduction, validation, and use of a discrimination index.

### Subgroup analysis

The results of the subgroup analysis according to the journal type and biomarker study design are shown in Table 3. Studies in clinical journal showed a trend for a higher RQS score than those in imaging journals (median 11.5 vs. 10), but this was not statistically significant. Studies in clinical journals (*n* = 20) were similar with those in imaging journals (*n* = 31) in most of the RQS score except for open science and data (*P* = .03). All three studies [4, 20, 47] made their code and/or data publicly available were published in the same clinical journal (*Neuro-Oncology*).

Prognostic/predictive studies showed a trend for a higher RQS score than diagnostic studies (median 12 vs. 10), but this was not statistically significant. Prognostic studies received a higher score than diagnostic studies in comparison with biologic correlates (*P* = .001) and comparison to a ‘gold standard’ (*P* < .001). Also, prognostic/predictive studies used calibration statistics (*P* = .02) and cut-off analysis (*P* = .001) more frequently than diagnostic studies, which is potentially useful for future modeling studies.

## Discussion

In this study, radiomics studies in neuro-oncologic imaging were evaluated in respect to the quality of both the science and the reporting, using radiomics quality score. Overall, radiomics studies still have room for improvement, with basic adherence rate of 37.1% out of total 16 items. In terms of protocol quality, radiomics studies were particularly deficient for testing image stability using test-retest analysis and a phantom study. Selected radiomics features were often correlated to non-radiomics features or biological phenotype, but linking them to clinical validation and achievement of clinical utility was insufficient. The radiomics models were often measured with discriminative statistics, while calibration statistics and cut-off analyses were underutilized. A high level of evidence for radiomics studies is critically lacking, with further limitations being demonstrated in their openness to data and code. Our results imply that a low quality of reporting may hamper the use of radiomics utilities as a tool for clinical decision-making, and several key domains in radiomics studies require significant improvement.

The six key domains used in this study were designed to support the integration of the RQS to facilitate the use in radiomics approaches. Adopted from the consensus statement of the FDA-NIH Biomarker Working Group [13], the three aspects of technical validation, biological/clinical validation, and assessment of cost-effectiveness for imaging biomarker standardization were included in domains 1, 3, and 5, respectively. With regards to technical validation, radiomics approaches are yet to become a reliable measure for the testing of hypotheses in clinical cancer research, with insufficient data supporting their precision or technical bias. Precision analysis using repeatability and reproducibility test was conducted in one study [10], but reproducibility needs to be tested using different geographical sites and different equipment. Furthermore, none of the evaluated studies reported analysis of technical bias using a phantom study, which describes the systemic difference between the measurements of a parameter and its real values [62]. According to our results, further technical validation needs to be achieved before radiomics analysis can be related to clinical outcomes.

Along with technical performance assessment, imaging biomarkers need to pass clinical performance assessment in multicenter studies before they can be considered for clinical use [13, 63]. For clinical validation, prospective testing of an imaging biomarker in clinical populations is required [64], but until now little studies have conducted a prospective study in the field of neuro-oncology. After biological/clinical validation, the cost-effectiveness of radiomics needs to be studied to ensure it provides good value for money compared with the other currently available biomarkers. However, no study has conducted cost-effectiveness analysis and only one study demonstrated net benefit improvement. Also, external validation is conducted in only 29.4% of the total studies. From the current standpoint, the clinical use of radiomics may seem far away, and technical and clinical validation is still required.

Subgroup analysis demonstrated that prognostic and predictive radiomics studies showed better quality than diagnostic studies in regard to comparison to ‘gold standard’, use of calibration statistics, and use of cut-off analysis. These are important for adoption of radiomics modeling, by demonstrating point estimates of prediction and actual data using calibration and by applying cut-off in the future studies. These measures further emphasize utility of radiomics modeling in clinic.

Biological validation of imaging biomarkers occurs relatively late in the development process [13]. In terms of biological validation, 74.5% of studies related radiomics features to biological correlates, such as the molecular subtype of IDH mutation or MGMT methylation status, while 62.7% of studies performed multivariate analysis using both radiomics and non-radiomic features. Nonetheless, biological validation of imaging-pathologic correlations [13] is not currently available for radiomics, as extensive spatial heterogeneity exists [1] and co-localized pathology data are currently not achievable. The unclear relationship with tumor biology is probably the reason why radiomics approaches have not influenced clinical decision-making or exhibited potential clinical utility.

It is important to adhere to the standardization of the radiomics features nomenclature and calculation according to the IBSI (International Biomarker Standardization Initiative) to improve reproducibility of scientific researches. Only 3 of studies made their code open, and many of studies did not provide detailed descriptions of the calculation of radiomics features and did not permit to clarify the details of radiomics calculation. Future studies are needed in terms of adherence to the standardization of radiomics features. Also, studies have utilized public data sources such as the TCIA did not made their own data publicly available. Further determination of the reproducibility of radiomics techniques requires collaborative multicenter studies, which would benefit greatly from the open availability of data and models.

Our study had several limitations. First, the publication of radiomics studies is not limited to the neuro-oncologic field or to MRI. Limits were placed on the search to permit in-depth analysis of applications for a particular disease where radiomics research seems to be most actively performed. Given the impact and number of populations of the selected studies, our results may actually represent a higher than average quality. Second, radiomics is still a developing imaging biomarker and the suggested RQS may be too ‘ideal’ to be qualified. The criteria of phantom study and multiple imaging acquisitions may become unrealistic in clinical situation. Also, segmentation stability earned score when it was performed by two readers or more, but recent development of deep learning segmentation may provide more robust result than manual segmentation. However, aiming for a higher level of evidence is necessary for the future use of radiomics approaches in future clinical trials and in the clinic.

## Conclusion

The quality of reporting of radiomics studies in neuro-oncology is currently not sufficient. Validation is necessary using external dataset, and improvements need to be made to feature reproducibility, analysis of the clinical utility, pursuits of a higher level of evidence in study design, and open science.

## Supplementary information

**Additional file 1: Table S1.** The six key domains of the radiomics quality score.

**Additional file 2: Figure S1.** RQS score according to the journal shows no definite systematic differences between the journals.

**Additional file 3: Table S2.** Characteristics of the included studies.

## Data Availability

All data are used in this study is provided in the electronic supplemental materials.

## Abbreviations

IDH: Isodehydroxygenase
LGG: Lower grade glioma
MGMT: O-6-methylguanine-DNA methyltransferase
MRI: Magnetic resonance imaging
RQS: Radiomics quality score
TRIPOD: Multivariable prediction model for Individual Prognosis OR Diagnosis
