# Supplementary material for: A systematic review reporting quality of radiomics research in neuro-oncology: toward clinical utility and quality improvement using high-dimensional imaging features
Source: BMC Cancer. 2020 Jan 10;20:29. doi: 10.1186/s12885-019-6504-5 (PMC6954557; doi:10.1186/s12885-019-6504-5)
Supplement: Supplementary file 1 — Additional file 1: Table S1. The six key domains of the radiomics quality score. [file 12885_2019_6504_MOESM1_ESM.docx]

**Table S1.** The six key domains of the radiomics quality score

| Domain |  |  | score | RQS criteria |
| --- | --- | --- | --- | --- |
| 1 | Image protocol quality | Well-documented image protocols (for example, contrast, slice thickness, energy, etc.) and/or usage of public image protocols allow reproducibility/replicability | + 1 (if protocols are well-documented)  + 1 (if public protocol is used) | 1 |
|  | Multiple segmentations | Segmentation by different physicians/algorithms/software, perturbing segmentations by (random) noise, segmentation at different breathing cycles. Analyse feature robustness to segmentation variabilities | + 1 | 2 |
|  | Phantom study on all scanners | Detect inter-scanner differences and vendor-dependent features. Analyse feature robustness to these sources of variability | + 1 | 3 |
|  | Imaging at multiple time points | Collect images of individuals at additional time points. Analyse feature robustness to temporal variabilities (for example, organ movement, organ expansion/ shrinkage) | + 1 | 4 |
| 2 | Feature reduction or adjustment for multiple testing | Decreases the risk of overfitting. Overfitting is inevitable if the number of features exceeds the number of samples. Consider feature robustness when selecting features | - 3 (if neither measure is implemented) + 3 (if either measure is implemented) | 5 |
|  | Validation | The validation is performed without retraining and without adaptation of the cut-off value, provides crucial information with regards to credible clinical performance | - 5 (if validation is missing)  + 2 (if validation is based on a dataset from the same institute)  + 3 (if validation is based on a dataset from another institute)  + 4 (if validation is based on two datasets from two distinct institutes)  + 4 (if the study validates a previously published signature)  + 5 (if validation is based on three or more datasets from distinct institutes) | 12 |
| 3 | Multivariable analysis with non-radiomics features | (for example, EGFR mutation) - is expected to provide a more holistic model. Permits correlating/inferencing between radiomics and non radiomics features | +1 | 6 |
|  | Detect and discuss biological correlates | Demonstration of phenotypic differences (possibly associated with underlying gene–protein expression patterns) deepens understanding of radiomics and biology | +1 | 7 |
|  | Comparison to ‘gold standard’ | Assess the extent to which the model agrees with/is superior to the current ‘gold standard’ method (for example, TNM-staging for survival prediction). This comparison shows the added value of radiomics | +2 | 13 |
|  | Potential clinical utility | Report on the current and potential application of the model in a clinical setting (for example, decision curve analysis). | +2 | 14 |
| 4 | Cut-off analyses | Determine risk groups by either the median, a previously published cut-off or report a continuous risk variable. Reduces the risk of reporting overly optimistic results | +1 | 8 |
|  | Discrimination statistics | Report discrimination statistics (for example, C‑statistic, ROC curve, AUC) and their statistical significance (for example, p‑values, confidence intervals). One can also apply resampling method (for example, bootstrapping, cross-validation) | + 1 (if a discrimination statistic and its statistical significance are reported) + 1 (if a resampling method technique is also applied) | 9 |
|  | Calibration statistics | Report calibration statistics (for example, Calibration-in‑the-large/slope, calibration plots) and their statistical significance (for example, *P*‑values, confidence intervals). One can also apply resampling method (for example, bootstrapping, cross-validation) | + 1 (if a calibration statistic and its statistical significance are reported) + 1 (if a resampling method technique is also applied) | 10 |
| 5 | Prospective study registered in a trial database | Provides the highest level of evidence supporting the clinical validity and usefulness of the radiomics biomarker | + 7 (for prospective validation of a radiomics signature in an appropriate trial) | 11 |
|  | Cost-effectiveness analysis | Report on the cost-effectiveness of the clinical application (for example, QALYs generated) | +1 | 15 |
| 6 | Open science and data | Make code and data publicly available. Open science facilitates knowledge transfer and reproducibility of the study | + 1 (if scans are open source) + 1 (if region of interest segmentations are open source) + 1 (if code is open source) + 1 (if radiomics features are calculated on a set of representative ROIs and the calculated features and representative ROIs are open source) | 16 |

**–**
