# Supplementary material for: A systematic review reporting quality of radiomics research in neuro-oncology: toward clinical utility and quality improvement using high-dimensional imaging features
Source: BMC Cancer. 2020 Jan 10;20:29. doi: 10.1186/s12885-019-6504-5 (PMC6954557; doi:10.1186/s12885-019-6504-5)

**Figure S1.** RQS score according to the journal shows no definite systematic differences between the journals.


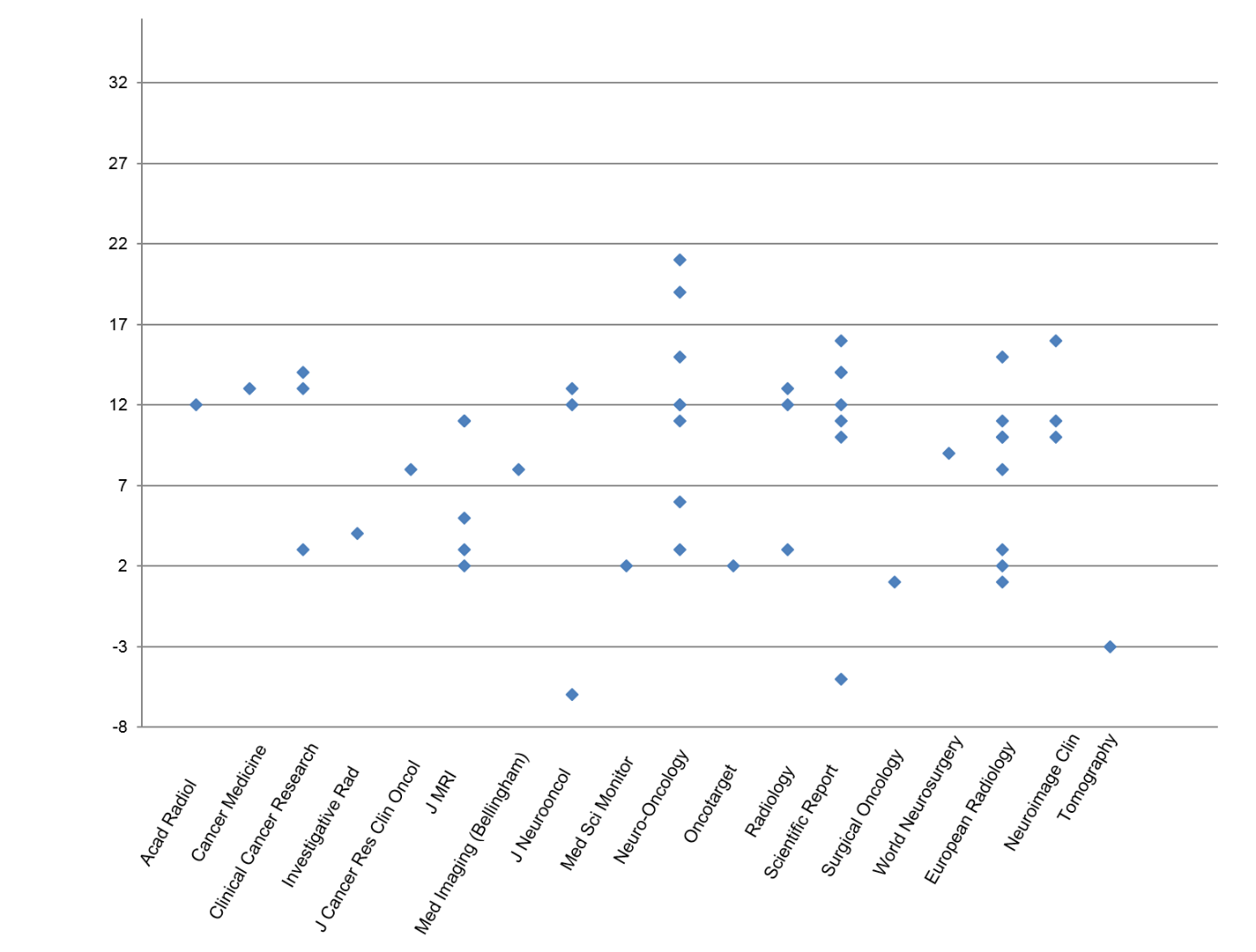

Supplement: Supplementary file 2 — Additional file 2: Figure S1. RQS score according to the journal shows no definite systematic differences between the journals. [file 12885_2019_6504_MOESM2_ESM.docx]
