# Supplementary material for: A systematic review reporting quality of radiomics research in neuro-oncology: toward clinical utility and quality improvement using high-dimensional imaging features
Source: BMC Cancer. 2020 Jan 10;20:29. doi: 10.1186/s12885-019-6504-5 (PMC6954557; doi:10.1186/s12885-019-6504-5)
Supplement: Supplementary file 3 — Additional file 3: Table S2. Characteristics of the included studies. [file 12885_2019_6504_MOESM3_ESM.docx]

**Table S2.** Characteristics of the included studies.

|  | **Author (year of publication)** | **Journal** | **Disease** | **Task** | **Intended Use of Biomarker** | **Number of patients (Training: Test)** | **External validation** | **Data Multicentricity**  **(Training)** |
| --- | --- | --- | --- | --- | --- | --- | --- | --- |
| 1 | Akbari H et al. (2018) | Neuro Oncology | Glioblastoma | Molecular classification | Diagnostic | 129 (75:54) | No | Single center, 3T |
| 2 | Arita H et al. (2018) | Sci Report | LGG | Molecular classification | Diagnostic | 169 (111: 58) | Yes | Multicenter, 1.5T and 3T |
| 3 | Artzi M et al. (2019) | J Magnetic Resonance Imaging | Glioblastoma, Metastasis | Differential Diagnosis | Diagnostic | 439 (351:88) | No | Single center, 3T |
| 4 | Artzi M et al. (2018) | J Magnetic Resonance Imaging | Glioblastoma, Vasogenic edema and Infiltration | Differential Diagnosis | Diagnostic | 102 (82: 20) | No | Single center, 3T |
| 5 | Bae S et al.  (2018) | Radiology | Glioblastoma | Survival | Prognostic | 217 (163:54) | No | Single center, 3T |
| 6 | Chen X et al.  (2019) | Academic Radiology | Glioblastoma | Survival | Prognostic | 127 (85:42) | No | Public, 1.5T or 3T |
| 7 | Ditmer A et al (2019) | J Neurooncology | Glioma | Grade | Diagnostic | 94 | No | Single center, 1.5T |
| 8 | Dong F et al. (2018) | European Radiology | Pilocytic astrocytoma and glioblastoma | Differential diagnosis | Diagnostic | 66 (44:22) | No | Two centers,1.0T/1.5T/3.0T |
| 9 | Eichinger P et al. (2017) | Sci Rep | LGG | Molecular classification | Diagnostic | 79 (59: 20) | Yes | Single center, 3.0T |
| 10 | Grossman P et al. (2017) | Neuro Oncology | Glioblastoma | Response to treatment | Predictive | 293 (126: 167) | Yes | Single center, 1.5 or 3.0T |
| 11 | Han Y et al. (2018) | J Neurooncol | LGG | Molecular classification | Diagnostic | 277 (184: 93) | No | Single center, 3.0T |
| 12 | Hsieh KL et al. (2017) | Oncotarget | Glioblastoma | Molecular classification | Diagnostic | 32 (32:0) | No | Public, 1.5T and 3T |
| 13 | Hu LS et al. (2017) | Neuro Oncology | Glioblastoma | Genomic Classification | Diagnostic | 48 (48: 0) | No | Single center, 3.0T |
| 14 | Ingrisch M et al. (2017) | Investigative Radiology | Glioblastoma | Survival | Prognostic | 66 (66:0) | No | Single center, 1.5 or 3.0T |
| 15 | Kang DS et al. (2018) | Neuro Oncology | PCNSL, Glioblastoma | Differential diagnosis | Diagnostic | 154 (112:42) | Yes | Single center, 3.0T |
| 16 | Kickingereder et al. (2016) | Radiology | Glioblastoma | Survival | Prognostic | 119 (79: 40) | No | Single center, 3.0T |
| 17 | Kickingereder et al. (2016) | Clin Cancer Research | Glioblastoma | Response to treatment | Predictive | 172 (112:60) | No | Single center, 3.0T |
| 18 | Kickingereder et al. (2016) | Radiology | Glioblastoma | Genomic Classification | Diagnostic | 152 (152:0) | No | Single center, 3.0T |
| 19 | Kickingereder et al. (2018) | Neuro Oncology | Glioblastoma | Survival | Prognostic | 181 (120: 61) | No | Single center, 3.0T |
| 20 | Kim JY et al. (2018) | Neuro Oncology | Glioblastoma | Differential diagnosis | Diagnostic | 118 (61: 57) | Yes | Single center, 3.0T |
| 21 | Lee MH et al. (2019) | World Neurosurgery | Glioblastoma | Molecular classification | Diagnostic | 123 (88:35) | Yes | Single center, 3.0T |
| 22 | Lao J et al. (2017) | Sci Rep | Glioblastoma | Survival | Prognostic | 102 (75:37) | Yes | Public, 1.5T or 3T |
| 23 | Li Q et al. (2017) | Sci Rep | Glioblastoma | Survival | Prognostic | 92 (60: 32) | Yes | Single center, 3.0T |
| 24 | Li Y et al. (2017) | J Neurooncology | LGG | Molecular classification | Diagnostic | 117 (78:39) | No | Single center, 3.0T |
| 25 | Li Y et al. (2018) | Eur Radiol | LGG | Molecular classification | Diagnostic | 186 (63: 123) | Yes | Public, 1.5T or 3T |
| 26 | Li Y et al. (2018) | Eur Radiol | LGG | Molecular classification | Diagnostic | 270 (200: 70) | No | Single center, 3.0T |
| 27 | Li Y et al. (2018) | Neuroimage Clinical | LGG | Molecular classification | Diagnostic | 272 (180: 92) | No | Single center, 3.0T |
| 28 | Li Z et al. (2017) | Sci Rep | LGG | Molecular classification | Diagnostic | 119 (60: 59 or 85: 34) | No | Single center, 3.0T |
| 29 | Li ZC et al. (2018) | Eur Radiol | Glioblastoma | Molecular classification | Diagnostic | 193 (133:60) | Yes | Public, 1.5T or 3T |
| 30 | Li ZC et al. (2018) | Cancer Med | Glioblastoma | Molecular classification | Diagnostic | 225 (118:107) | Yes | Public, 1.5T or 3T |
| 31 | Liu X et al. (2018) | Neuroimage Clinical | LGG | Survival | Prognostic | 300 (216:84) | Yes | Public, 1.5T or 3T |
| 32 | Liu TT et al. (2017) | Neuro Oncology | Glioblastoma | Response to treatment, Survival | Prognostic, Predictive | 117 (68: 79) | Yes | Public, 1.5T or 3T |
| 33 | Liu Z et al. (2017) | Neuroimage Clinical | LGG | Epilepsy prediction | Predictive | 286 (194: 92) | No | Single center, 3.0T |
| 34 | Lu CF et al. (2018) | Clin Cancer Research | Glioma | Molecular classification | Diagnostic | 284 (214: 70) | Yes | Public, 1.5T or 3T |
| 35 | Lohmann P et al. (2018) | Sci Report | Glioma | Molecular classification | Diagnostic | 84 | No | Single center, 3.0T |
| 36 | McGarry SD et al. (2016) | Tomography | Glioblastoma | Survival | Prognostic | 81 (81:0) | No | Single center, 1.5 or 3.0T |
| 37 | Prasanna P et al. (2018) | Eur Radiol | Glioblastoma | Survival | Prognostic | 65 (65: 0) | No | Public, 1.5T or 3T |
| 38 | Qin JB et al. (2017) | Medical Science Monitor | Gliomas | Grade | Diagnostic | 66 (66:0) | No | Single center, 3.0T |
| 39 | Ren Y et al. (2019) | J Magn Reson Imaging | LGG | Molecular classification | Diagnostic | 105 | No | Single center, 3.0T |
| 40 | Sanghani P et al. (2018) | Surg Oncol | Glioblastom | Survival | Prognostic | 163 | No | Public, 1.5T or 3T |
| 41 | Su C et al. (2018) | Eur Radiol | Gliomas | Grade | Diagnostic | 220 | No | Single center, 3.0T |
| 42 | Rathore S et al. (2018) | Sci Rep | Glioblastoma | Molecular classification, survival | Diagnostic, Prognostic | 261 (208:53) | No | Single center, 3.0T |
| 43 | Suh HB et al. (2018) | Eur Radiol | PCNSL, Glioblastoma | Differential diagnosis | Diagnostic | 77 (77:0) | No | Single center, 3.0T |
| 44 | Tian Q et al. (2018) | J Magn Reson Imaging | Glioma | Grade | Diagnostic | 153 (153: 0) | No | Single center, 3.0T |
| 45 | Wang Q et al. (2019) | J Magn Reson Imaging | Glioma | Grade | Diagnostic | 84 (56: 29) | No | Single center, 1.5T |
| 46 | Wu S et al. (2019) | J Cancer Res Clinic Oncology | LGG | Molecular classification | Diagnostic | 126 (84: 42) | No | Public, 1.5T or 3T |
| 47 | Xi YB et al. (2018) | J Magn Reson Imaging | Glioblastoma | Molecular classification | Diagnostic | 118 (98: 20) | Yes | Single center, 3.0T |
| 48 | Yu J et al. (2017) | Eur Radiol | LGG | Molecular classification | Diagnostic | 140 (110: 30) | No | Single center, 3.0T |
| 49 | Zhang X et al. (2018) | J Magn Reson Imaging | LGG | Molecular classification | Diagnostic | 103  (73:30) | No | Public, 1.5T or 3T |
| 50 | Zhou H et al. (2017) | Neuro Oncology | LGG | Molecular classification, Survival | Diagnostic, Prognostic | 165  (165:0) | No | Public, 1.5T or 3T |
| 51 | Zinn PO et al. (2019) | Clin Cancer Research | Glioblastoma | Molecular classification | Diagnostic | 93 | No | Public, 1.5T or 3T |

Abbreviations: LGG-lower grade glioma; PCNSL – primary central nervous system lymphoma
